# Predictive and Prognostic Biomarkers and Tumor Antigens for Targeted Therapy in Urothelial Carcinoma

**DOI:** 10.3390/molecules29081896

**Published:** 2024-04-22

**Authors:** Aditya Eturi, Amman Bhasin, Kevin K. Zarrabi, William J. Tester

**Affiliations:** 1 Department of Medical Oncology, Sidney Kimmel Cancer Center, Thomas Jefferson University, Philadelphia, PA 19107, USA; kevin.zarrabi@jefferson.edu (K.K.Z.); william.tester@jefferson.edu (W.J.T.); 2 Department of Internal Medicine, Thomas Jefferson University, Philadelphia, PA 19107, USA; amman.bhasin@jefferson.edu

**Keywords:** urothelial carcinoma, bladder cancer, biomarkers, targeted therapies, ctDNA

## Abstract

Urothelial carcinoma (UC) is the fourth most prevalent cancer amongst males worldwide. While patients with non-muscle-invasive disease have a favorable prognosis, 25% of UC patients present with locally advanced disease which is associated with a 10–15% 5-year survival rate and poor overall prognosis. Muscle-invasive bladder cancer (MIBC) is associated with about 50% 5 year survival when treated by radical cystectomy or trimodality therapy; stage IV disease is associated with 10–15% 5 year survival. Current therapeutic modalities for MIBC include neoadjuvant chemotherapy, surgery and/or chemoradiation, although patients with relapsed or refractory disease have a poor prognosis. However, the rapid success of immuno-oncology in various hematologic and solid malignancies offers new targets with tremendous therapeutic potential in UC. Historically, there were no predictive biomarkers to guide the clinical management and treatment of UC, and biomarker development was an unmet need. However, recent and ongoing clinical trials have identified several promising tumor biomarkers that have the potential to serve as predictive or prognostic tools in UC. This review provides a comprehensive summary of emerging biomarkers and molecular tumor targets including programmed death ligand 1 (PD-L1), epidermal growth factor receptor (EGFR), human epidermal growth factor receptor 2 (HER2), fibroblast growth factor receptor (FGFR), DNA damage response and repair (DDR) mutations, poly (ADP-ribose) polymerase (PARP) expression and circulating tumor DNA (ctDNA), as well as their clinical utility in UC. We also evaluate recent advancements in precision oncology in UC, while illustrating limiting factors and challenges related to the clinical application of these biomarkers in clinical practice.

## 1. Introduction

Urothelial carcinoma can arise throughout the genitourinary tract, most commonly in the bladder. For patients with locally advanced or metastatic disease, survival outcomes remain poor. Recent clinical trials have shown benefits from emerging treatments in the neoadjuvant, adjuvant and metastatic disease settings. These trials include the application of chemotherapy, immunotherapy and targeted therapy for selected patient populations. However, the level of benefit has been modest; only a minority of patients chosen for a specific therapy derive significant benefit for most of these new therapies. With current treatment selection practices, an individual patient may undergo treatment with limited efficacy and significant potential toxicity. Unfortunately, the predictive value of current biomarkers to tailor treatment selection remains modest [1,2]. There are few biomarkers approved for use in the clinical setting. Biomarkers currently used in other cancers are under review in urothelial cancers and could potentially lead to adoption of therapies such as HER2-directed drugs and PARP inhibitors. None of the current biomarkers with regulatory approval for other malignancies have been validated for prognostic benefit in UC and are not employed in patient care.

A more rational application of modern therapies should harness cellular markers and patient characteristics to improve patient outcomes. It is also likely that a better understanding of biomarkers will also lead to the development of additional therapeutics. To improve clinical decision making, there remains an unmet need to develop better molecular or genetic markers with predictive value for the risk of disease recurrence or progression.

Newer biomarkers that have been developed hold promise as either predictive and/or prognostic tools. Potentially useful biomarkers include PD-L1, tumor mutational burden, DNA repair mutations, excision repair cross-complementing protein 1 (ERCC1) and excision repair cross-complementing protein 2 (ERCC2), FGFR mutation, EGFR expression or mutation, HER2 expression, PARP expression and circulating tumor DNA (ctDNA). Molecular markers can be measured in tumor tissue or blood. Moreover, early studies also suggest value in the measurement of urinary markers in patients with early-stage urothelial carcinoma. This review will summarize evidence regarding the value of current and emerging biomarkers that may be of clinical utility in the selection treatment for individual patients. Clinically useful markers will be those that ultimately provide prognostic or predictive capacity beyond that offered by conventional clinical and histologic parameters.

## 2. Programmed Death Ligand-1

Programmed death ligand-1 (PD-L1) is a promising companion biomarker used to predict benefit for patients with several types of cancer treated with immunotherapy. Unfortunately, a high level of benefit has not been realized for most urothelial carcinoma patients. Although immunotherapy has established a role in the treatment paradigm of advanced urothelial carcinoma, there does not appear to be a strong predictive value for PD-L1 testing in these patients [3]. PD-L1 is not consistently predictive, and the correlation of its expression is not strong due to variable data. Challenges with PD-L1 testing include standardization of testing with a variety of assays and antibodies for that immunohistochemical assessment that is available and their weak predictive value for response rates to immunotherapy regardless of level of expression. Currently, there are several FDA-approved immunohistochemical (IHC) assays for PD-L1 expression in urothelial carcinoma: Ventana SP142 (Atezolizumab), Ventana PD-L1 SP263 (Durvalumab), 22C3 pharmDx (Pembrolizumab) and 28-8 pharmDx (Nivolumab). Each test has variance in methods (immune cell area score, combined positive score and tumor cell area score) and cutoffs for reporting positive scores. Staining intensity has also been reported as variable between tests with Ventana testing having deeper staining profiles [4].

For example, IMvigor210 was a Phase 2 trial exploring the efficacy and safety of atezolizumab in 119 patients with metastatic or locally advanced urothelial carcinoma and who were considered to be platinum-ineligible. In the population of 119 patients, 67% showed some level of PD-L1 expression. Approximately 20% of patients were PD-L1 non-expressors, and response rates were similar between PD-L1(+) and PD-L1(−) subgroups [5].

CheckMate 274 was a pivotal phase 2 trial that established the value of adjuvant nivolumab in the treatment algorithm for patients with muscle-invasive urothelial carcinoma following radical surgery. The majority of the patients in the trial had PD-L1 expression less than 1%, and 40% of enrolled patients were found to have PD-L1 expression ≥ 1%. The intent-to-treat population was randomized in a 1:1 fashion to receive either nivolumab or intravenous placebo. Patients were stratified by PD-L1 expression of ≥1% or <1%. A significant benefit of adjuvant immunotherapy was reported in both the intention-to-treat population and in patients with a PD-L1 expression level of 1% or more. Patients with expression ≥ 1% were found to achieve improved disease-free survival at both 6 and 12 months with a hazard ratio of 0.55 when treated with nivolumab [6]. Overall survival data for CheckMate 274 have not matured at the time of this review; therefore, we cannot conclude whether PD-L1 expression impacted overall survival [3]. The AMBASSADOR trial was another phase 3 randomized adjuvant study that evaluated the administration of checkpoint inhibitor for patients with muscle-invasive urothelial cancer. This trial reported the benefit of adjuvant pembrolizumab in being disease-free, but not in overall survival. The presence of PD-L1 positivity using the standard CPS score was associated with a better prognosis, but it was not predictive of a pembrolizumab benefit. The authors concluded that PD-L1 status should not be used to select patients for adjuvant treatment [7].

The phase Ib JAVELIN Solid Tumor study evaluated the use of second-line treatment with Avelumab. This trial enrolled 242 evaluable patients with metastatic urothelial carcinoma who had progressed on one line of treatment prior to receiving avelumab. Patients with PD-L1 expression ≥ 5% showed an improved objective response rate (ORR) and overall survival (OS) compared to those with lower levels of PD-L1 expression [8].

The JAVELIN BLADDER 100 trial evaluated the role of maintenance avelumab in 700 patients who completed first-line platinum-based chemotherapy and were randomly assigned to receive avelumab or best supportive care. In the intent-to-treat population, the 1-year survival was improved in the group that received avelumab (71.3% versus 58.4%, hazard ratio for death 0.69; 95% confidence interval [CI], 0.56 to 0.86; *p* = 0.001). In the PD-L1-positive population, the 1-year survival was also improved in the patients treated with avelumab compared to the control (79.1% versus 60.4%; hazard ratio for death 0.56; 95% CI, 0.40 to 0.79; *p* < 0.001). In this maintenance trial, the overall survival benefit with avelumab as compared with the control was somewhat greater in the PD-L1-positive population than in the overall population, but the result was significant in both populations. Furthermore, in the control group, the median overall survival was longer in the PD-L1-positive population than in the overall population (17.1 versus 14.3 months), suggesting that PD-L1 expression may not be an indicator of worse prognosis in a population of patients who achieve disease control with chemotherapy [9].

## 3. Tumor Mutational Burden—TMB

Tumor mutational burden (TMB) is remarkably high within urothelial carcinoma, ranking third behind lung cancer and melanoma [10]. TMB had been traditionally analyzed using whole-exome sequencing; however, in clinical practice next-generation sequencing has been used as an alternative to whole-exome sequencing. Studies assessing The Cancer Genome Atlas for mutations in bladder cancer aimed to identify actionable targets. In this cohort analysis, TMB demonstrated predictive value in predicting the response to immunotherapy [11]. High TMB results in an increase in neo-antigens, increasing the potential for a response to reactivated T-cells with checkpoint blockade. The presence of increased neoantigens allows for a more robust immune recognition and response as a result of immunotherapy [12]. A real-world example of TMB reflecting a potential predictive marker in urothelial cancer was seen in a retrospective analysis conducted on VA patients. This retrospective analysis of urothelial cancer treated with checkpoint inhibitors found that those with high-TMB tumors had median overall survival of 19.8 months, in comparison to that of 14.2 months for those with low-TMB ones [13]. While this study showed predictive value in the setting of a response to checkpoint inhibitors, this study also suggests that TMB may be independently prognostic in UC.

Several trials further describe and evaluate the predictive value of TMB, specifically related to immunotherapy response. IMvigor 210 was a trial evaluating atezolizumab in urothelial cancer patients who were platinum-ineligible or had progressed on platinum-based chemotherapies looking primarily at overall response rates. This study also included exploratory analysis looking at the effect of tumor mutational burden. Cohort 1 enrolled patients who had metastatic disease but were cisplatin-ineligible. Cohort 2 enrolled those with inoperable locally advanced or with metastatic disease and progression after platinum-based chemotherapy. Tumor mutational load was assessed via Foundation Medicine assay. The median mutational load was found to be increased in responders versus in non-responders (12.4/Mb versus 6.4/Mb) (*p* < 0.0001) [14]. In addition, patients with the highest mutational burden experienced the longest overall survival [5]. CheckMate 275 assessed nivolumab in a similar population of patients with metastatic or surgically unresectable urothelial carcinoma. Of the 270 patients treated, 139 had viable tissue for the determination of TMB status; 47 patients were included in the high-TMB expression tertile. Compared to the entire 139-patient population, those in the high-TMB expression group were found to have n improved ORR (31.9 versus 21.6%), PFS (3.5 versus 1.9 months) and OS (11.6 versus 7.2 months) [3].

PURE-01 is a phase 2 trial evaluating neoadjuvant pembrolizumab in MIBC patients. As for the two other trials described above, PURE-01 also assessed pre-surgical tissue for TMB. The TMB level was found to correlate with the response to immunotherapy. Those with higher TMB were more likely to be found to have a pT0 response at the time of cystectomy. These authors reported that a cutoff value of TMB 15 mut/MB was predictive of a pT0 response [15].

Some of the challenges of translating TMB for use in predicting the response to immunotherapy revolve around the assessment of TMB. Each other study described had a different categorization for TMB (categorical vs. ordinal vs. continuous variables). The standardization and consistent investigation of TMB would better allow for TMB to be a clinically useful predictive marker in UC. Much like PD-L1 expression, TMB has multiple FDA-approved assays that utilize next-generation sequencing (NGS) [16]. TMB has good potential for predictive value in patients being considered for checkpoint inhibitors. Ultimately, the further study of PD-L1 with relation to TMB would help determine which biomarker should dictate treatment with checkpoint inhibitors. TMB has not been validated for predictive value, though it remains readily accessible with existing commercial assays. In this way, TMB is further along than other biomarkers in the potential to help guide clinical decision making.

## 4. DNA Damage Repair Pathway Mutations

Biomarkers of DNA damage repair (DDR) pathways have been inconsistently reported to have prognostic or predictive value for patients with urothelial cancer. Some phase 2 trials report that the presence of DDR mutations imparts an increased susceptibility to cisplatin-based chemotherapy. However, other series found worse overall survival associated with some DDR mutations. Commonly reported gene mutations relevant to urothelial cancer include the following: Ataxia-telangiectasia mutated gene (*ATM*), retinoblastoma 1 (*RB1*), FA Complementation Group C (*FANCC*), excision repair cross-complement group 2 (*ERCC2*) and meiotic recombination 11 (*MRE11*). One retrospective study taken from three independent cohorts of patients with relapsed or advanced UC found that 165/302 (55%) patients harbored a DDR mutation. This study found that the presence of the *ATM* mutation was associated with worse OS while other DDR mutations were associated with improved survival [17].

**Figure 1:**
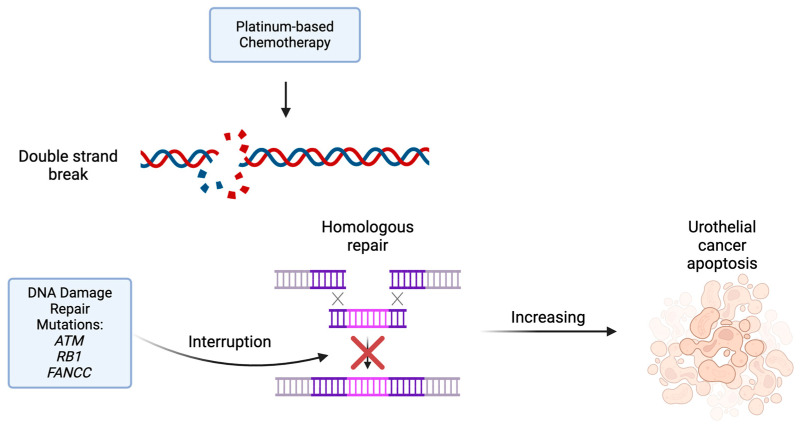
Platinum-based chemotherapy creates double-strand DNA breaks leading to cancer cell apoptosis. Tumors with DNA damage repair mutations have an interruption in DNA repair mechanisms such as homologous repair resulting in increasing cancer cell apoptosis and death.

The predictive value of DDR mutations has proven complex. These pathways are implicated in tumor progression and treatment response. These genes are inherently part of DNA repair mechanisms. The presence of DDR mutations has been associated with a platinum response in the neoadjuvant setting. Platinum chemotherapy is associated with double-strand breaks. Therefore, in tumors with an excess of DDR mutations, they are particularly sensitive to platinum-based chemotherapy as they lack the mechanisms for DNA repair (Figure 1) [18]. A cohort of patients in a phase II clinical trial with *ATM*, *RB1* and *FANCC* alterations treated with cisplatin-based neo-adjuvant chemotherapy experienced longer overall and disease-specific survival compared to patients without these mutations. Patients with at least one mutation were noted to have an 85% 5-year survival, compared to 46% for patients without mutation in these genes [19]. This finding suggests that the *ATM* gene may be a biomarker for poor prognosis overall, except for patients treated with neoadjuvant cisplatin, who experience improved OS.

Other trials have also studied the predictive value of these genomic markers in relation to clinical response. Two such trials, RETAIN and HCRN GU 16-257, incorporated the presence of DDR mutations in protocol-specified decision making and evaluated clinical outcomes. In the phase 2 RETAIN trial, patients with at least one DDR mutation and no cystoscopic evidence of disease following neoadjuvant cisplatin-based chemotherapy were monitored and did not undergo immediate cystectomy. Patients with tumors harboring the *RB1* mutation were found to have the highest rate of recurrence at 61% [20]. At a 2-year follow-up, the metastasis-free survival of patients with at least one DDR mutation was 76.9%, similar to that of the group who received bladder-directed therapy [21]. HCRN GU 16-257 used a similar paradigm, sequencing the post-TURBT specimen for DDR mutations; however, *ATM*, *RB1* and *FANCC* were not associated with a clinical response [22]. These mutations were, however, associated with longer bladder-intact MFS [23]. These mutations may eventually allow for the risk stratification and selection of patients who can proceed with active surveillance in a bladder-sparing fashion while concurrently identifying patients who may need upfront cystectomy.

There are similar challenges in predicting the treatment response for patients selected for bladder-preserving trimodality therapy. The current clinical criteria to select patients for bladder preservation remain insufficient. Like *ATM*, *FANCC* and *RB1*-*MRE11* have been candidate predictive biomarkers for radiosensitivity. *MRE11* is a DNA nuclease involved in responding to DNA damage, specifically double-strand breaks and stalled replication forks [24,25]. One study looking at *MRE11* in pooled patients from trimodality therapy trials found that higher *MRE11* signal ratios were associated with lower disease-specific mortality [26].

Alterations in DDR pathway genes may also predict the clinical benefit for patients treated with PD-1 and PD-L1 inhibitors. Pretreatment biopsy specimens from 60 patients treated with anti-PD-1 or PD-L1 antibodies underwent tumor sequencing by the MSK_IMPACT assay and the presence of DDR alterations was correlated with clinical outcomes. Known or likely deleterious DDR mutations were identified in 28 (47%) and 15 (25%) patients, respectively. The objective response rate was higher for patients whose tumors contained any DDR alteration than for those whose tumors were wild-type (67.9% v 18.8%; *p* < 0.001). This study also found that tumor DDR status was of greater predictive value than mutational load in predicting response, progression-free survival and overall survival [27]. The finding of DDR mutations in pretreatment biopsy specimens has not yet been validated for clinical decision making, but, at the present time, it seems more promising than the measurement of tumor mutational load or PD-L1 level.

## 5. ERCC1 and ERCC2

Excision repair cross complementing (ERCC) is an integral component in the nucleotide excision repair (NER) system. The cytotoxic effects of chemotherapy, in particular platinum-based therapy, are based in part on the formation of DNA platinum complexes. NER confers resistance to platinum-based chemotherapy by clearing these complexes. Increased tissue expression of ERCC1 mRNA has been associated with platinum resistance in several cancers. A study that evaluated ERCC1 mRNA expression in patients with advanced UC treated with cisplatin-based chemotherapy reported that the median survival of patients with low expression was significantly better than those with high expression [28].

A meta-analysis of nine randomized adjuvant trials including 945 patients reported improved overall and disease-free survival for patients treated by radical cystectomy and adjuvant cisplatin chemotherapy compared to those who underwent radical cystectomy alone. The pooled hazard radio for overall survival was 0.77 (*p* = 0.049) and for disease-free survival was 0.66 (*p* = 0.014) [29]. A subsequent retrospective study of adjuvant cisplatin suggested that ERCC1 had both prognostic and predictive value in patients who underwent cystectomy for UC. ERCC1 expression by immunohistochemistry was analyzed in patients treated with cystectomy plus adjuvant cisplatin chemotherapy and compared to patients treated with cystectomy alone. Among 93 patients, ERCC1 expression was positive in 54 (58%) and negative in 39 (42%). ERCC1 positivity was significantly associated with longer survival in the group who did not receive chemotherapy. In the group who did receive chemotherapy, ERCC1 positivity was associated with shorter disease-free and overall survival [30]. An alternative retrospective study of patients with bladder cancer evaluating biological correlations with ERCC1, however, did not confirm these findings [31]. The value of ERCC1 as a predictive marker is presently uncertain. Future prospective, randomized studies are needed to establish its value in the selection of patients who are likely to benefit from adjuvant chemotherapy.

ERCC2 is another gene associated with the NER system which has more clarity in the body of data pertaining to gene mutation status and cisplatin sensitivity. Like ERCC1, higher ERCC2 expression conferred cisplatin resistance in pre-clinical models [32]. Whole-exome sequencing was performed on 50 patients who received neoadjuvant cisplatin-based chemotherapy followed by cystectomy. Patients with ERCC2 mutations in pretreatment tumor biopsy specimens were more likely to achieve a complete pathologic response to neoadjuvant cisplatin than patients without ERCC2 mutations. Furthermore, any amount of ERCC2 mutations resulted in an increased response to cisplatin. This finding suggests that there may be a haplo-insufficient effect of ERCC2 mutations [33]. Further studies have attempted to validate this effect—looking at patients who received neoadjuvant cisplatin and then correlating ERCC2 expression with pathologic response following resection. A retrospective study of 165 patients with muscle-invasive bladder cancer treated with neoadjuvant chemotherapy and radical cystectomy analyzed clinical outcomes related to pretreatment ERCC2 status. This study found that the presence of the ERCC2 mutation correlated with the pathologic response to cisplatin neoadjuvant therapy, but it was not associated with an improvement in recurrence-free or overall survival [34]. As with ERCC1, ERCC2 needs further validation and assessment in prospective cohorts to determine its use clinically. Both appear to have predictive value, though ERCC2 appears more promising at this time and neither of them have been validated for standard clinical use yet.

## 6. Fibroblast Growth Factor Receptor—FGFR

FGFR is an important growth factor receptor integral to carcinogenesis; it supports tumor growth by promoting angiogenesis and the regeneration of tissue. Urothelial cancers have a particularly high incidence of mutations or alterations in the FGFR. Four distinct FGFR subtypes have been implicated in urothelial cancer. These molecules are membrane-bound tyrosine kinase receptors involved in cellular proliferation, differentiation and steroid synthesis [35]. The incidence of FGFR3 alterations or mutations vary, depending on tumor stage, and occur in up to 80% of stage Ta tumors and are associated with favorable outcomes In stage T2 and greater tumors, FGFR3 mutations are less common (10–20%) [36]. One retrospective study of 72 patients with bladder cancer reported the presence of FGFR3 mutations in 64% of pTa bladder cancers and none in higher stage tumors [37]. A cohort study using the Flatiron Health database isolated a group of patients that would be eligible for erdafitinib based on recent FDA approval. In this database 343/761 patients were reported to have FGFR testing. In this 343-patient subpopulation, 71 (20.7%) were found to have either an FGFR3 alteration or FGFR2/3 fusion [38].

A higher incidence of FGFR3 alterations has been found in patients with upper-tract as opposed to lower-tract tumors [39]. PROOF 302 was a phase 3 clinical trial of adjuvant infigratinib for patients with either upper- or lower-tract UC. Although the trial was closed early and is not informative regarding therapy, it did yield important insight into FGFR alterations. Of the 617 patients with evaluable genomic data, 188 (30%) had alterations in FGFR 1–4 genes, 43% in upper tract, 23% in MIBC and 9% with unknown origin UC. FGFR3 alterations were found in 19% overall, 30% with upper tract, 13% with bladder and 9% with unknown tumor site [40]. For inhibition of the FGFR, either specific receptors or non-specific inhibition is currently under investigation in multiple trials—either as a single agent or in combination with other therapies [41].

**Figure 2:**
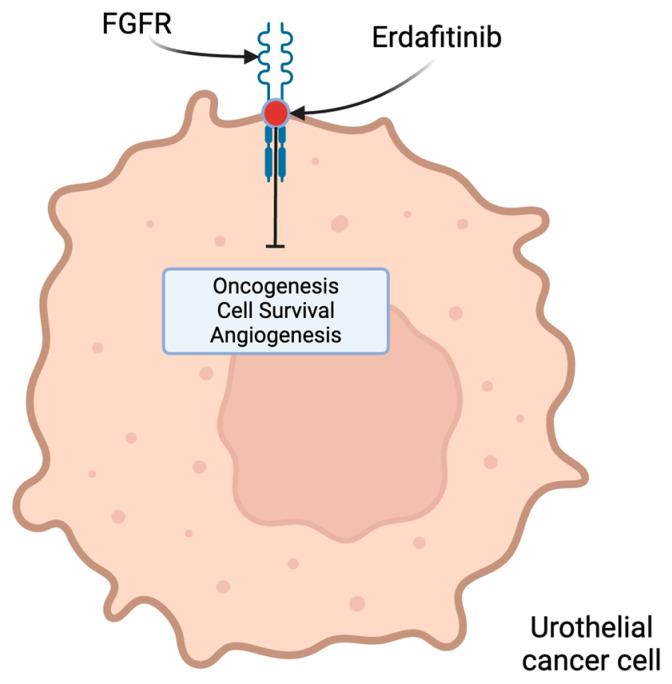
FGFR as a therapeutic target for pan-FGFR-sensitive inhibitor Erdafitinib inhibiting oncogenesis, survival and angiogenesis resulting in increased cell death.

Erdafitinib, a pan-FGFR inhibitor, is the only agent approved for patients with metastatic or advanced UC at the present time. As an FGFR inhibitor, erdafitinib results in increased cell death by preventing oncogenesis, survival and angiogenesis (Figure 2). In the BCL2001 trial, patients previously treated with chemotherapy or ineligible for cisplatin and had an FGFR 3 mutation or FGFR 2/3 fusions were included. Erdafitinib treatment produced a 40% response rate, resulting in FDA approval in this setting [42]. Clinical trials such as the NORSE study have evaluated the efficacy of erdafitinib combined with ICI [43]. Other trials, including THOR, evaluated erdafitinib versus chemotherapy following the failure of ICI in patients with select FGFR 2/3 mutations or fusions. This trial demonstrated superior survival, progression-free survival and response rates for erdafitinib compared to for single-agent chemotherapy in patients with FGFR 2/3 alterations [44]. Updated data from THOR showed that erdafitinib treatment achieved its primary endpoint, improving overall survival compared to chemotherapy (median OS 12.1 vs. 7.8 months, HR 0.64, *p* = 0.005) [45]. A phase II/III trial looking at novel FGFR inhibitor, rogaratinib, versus chemotherapy in patients specifically with FGFR1/3 mRNA overexpression showed mixed results—the overall study did not show a significant difference in OS, the ORR, or PFS. Looking at the high FGFR alteration expressors, however, showed a significantly higher ORR compared to chemotherapy (52.4% vs. 20.7%) [46]. There may be a role for future studies evaluating the dual inhibition of ERBB3 and FGFR3 simultaneously as this may be a mechanism of tumor resistance to FGFR inhibitors [47]. These trials show that the FGFR may be a helpful predictive biomarker, specifically if combined with appropriate therapies; however, currently the picture is equivocal as the treatment landscape with FGFR inhibitors is relatively novel.

## 7. Human Epidermal Growth Factor Receptors Inhibitors: HER2 and EGFR (HER1)

**Figure 3:**
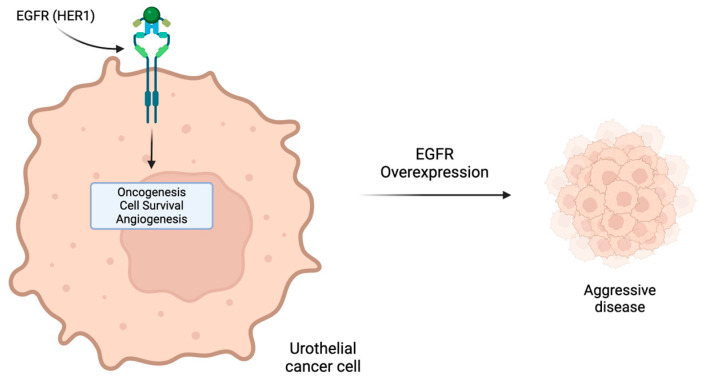
EGFR (HER1) as a potential therapeutic target. Overexpression of EGFR in urothelial cancer cells is a poor prognostic marker associated with more aggressive disease.

The HER growth factor receptor family includes cell surface proteins that have been strongly implicated in carcinogenesis, cell proliferation, invasion and metastasis [48,49,50]. HER1 (EGFR/ErbB-1) and HER2 (ErbB-2) transmembrane receptors play pivotal roles in the regulation of proliferation in UC, offering potential targets for directed therapy [51,52]. However, the value of these biomarkers remains controversial partly due to the heterogeneity of expression between histologic subtypes of UC and low concordance between the ERBB2 protein level and gene amplification in urothelial carcinomas [52,53]. For example, Hansel et al. reported HER2 overexpression (IHC) in 36% but genomic amplification in only 10% of tumors [54]. Some studies report that overexpression has been associated with clinically aggressive disease and poor prognosis (Figure 3) [55,56,57]. In patients with muscle-invasive bladder cancer, the incidence of HER1 overexpression is estimated at 55%, while HER2 overexpression is estimated at 37% [58].

Currently, no therapeutic agents are approved for UC patients whose tumors overexpress HER1 or HER2. Phase II trials of trastuzumab, a monoclonal antibody to HER2, added to conventional chemotherapy has shown limited benefit, while revealing significant cardiotoxicity. The single-agent activity of monoclonal antibodies or oral small-molecule inhibitors of HER2 are associated with overall response rates of less than 10% [59,60,61]. The multicenter phase II MyPathway study demonstrated the safety of trastuzumab when combined with pertuzumab in a dual anti-HER2 treatment approach; however, the efficacy of this treatment in UC remains to be determined by large prospective trials [62]. A randomized phase II trial reported the results of 88 patients treated with gemcitabine/cisplatin with or without cetuximab, a HER1-directed agent. The overall response rate with gemcitabine/cisplatin cetuximab was 61%, not significantly different from the gemcitabine/cisplatin arm. In addition, no significant difference in progression-free or overall survival was found. However, a greater risk of grades 3 and 4 adverse events, including thromboembolic events, occurred with the addition of cetuximab to chemotherapy [63].

Oral tyrosine kinase inhibitors (TKIs) have also been evaluated for patients with UC tumors that overexpress HER1 (EGFR) and HER2 in phase I, II and III clinical trials. A trial of lapatinib (anti-HER2) demonstrated that only 1 of 34 evaluable patients achieved a partial response. However, the increased expression (2+ or 3+) of either HER1 or HER2 was associated with improved overall survival, suggesting the possibility of benefit in the biomarker-positive subgroup [64]. A phase III trial screened 446 patients with metastatic UC. Fifteen percent of patients were negative for HER1 and HER2, 39% were positive for HER1 only, 13% for HER2 only and 33% for HER1 and HER2. No significant difference was found in OS in terms of HER status in the screened population, suggesting that the expression of HER1 or HER2 is not of prognostic significance. The biomarker-positive patients were randomly assigned for treatment with lapatinib maintenance or placebo post-chemotherapy. No improvement in progression-free or overall survival was seen with the addition of lapatinib maintenance for the biomarker-positive population. These studies demonstrated that lapatinib as a monotherapy was associated with a minimal response rate and did not improve outcomes in HER1/2-positive patients [65].

Phase II studies, including the LUX-Bladder 1 trial (NCT02780687) and NCI-MATCH EAY131 basket trial, evaluated treatment with afatinib, a pan-HER TKI, and demonstrated mixed results in patients with HER2/3 mutations, while no responses were seen in 42 patients expressing HER1 (EGFR) mutations [59,65,66,67]. An investigation into the efficacy of an alternative pan-HER TKI, neratinib, studied in the SUMMIT basket trial reached similar conclusions with no responses seen in HER2/3 UC patients [68].

The clinical benefit of HER1/2-directed monoclonal antibodies and tyrosine kinase inhibitors in the management of patients with UC has been disappointing to date. However, novel treatment strategies with anti-HER2/3 antibody–drug conjugates (ADC) appear more promising. ADCs are composed of monoclonal antibodies that target HER2 receptors and are chemically linked to a cytotoxic agent. Disitamab vedotin (RC48-ADC) was evaluated in two phase II studies with a total of 107 patients with HER2-positive (IHC 2+ or 3+) tumors, previously treated with at least one line of prior therapy. The overall response rate was 51% and demonstrated a favorable safety profile [69]. The combination of disitamab vedotin with toripalimab, an anti-PD-1 checkpoint inhibitor, elicited an objective response of 80% in a 14-patient phase II trial [70]. Despite the overall response, the durability of the response has been an issue with ADCs. Several phase I and II clinical trials exploring the potential of ADCs are currently ongoing (NC04632992, NCT04602117, DESTINY-PanTumor02, NCT04639219, MyTACTIC and NCT03523572), as more research is necessary to determine the potential therapeutic benefits of ADC pharmacology in the treatment of UC [71]. If larger confirmatory clinical trials show that these compounds produce improved efficacy, then the measurement of HER expression may achieve predictive value.

## 8. Poly (ADP-Ribose) Polymerase (PARP)

PARP expression or activity has the potential to serve as both a prognostic and predictive biomarker for patients with UC. PARP plays a significant role in the repair of platinum-induced DNA damage and its overexpression leads to reduced sensitivity to platinum-based chemotherapy. In human urothelial cancer cell lines and xenograft models, cells were treated with cisplatin, a PARP inhibitor (PARPi) or both. In a xenograft mouse model, a reduced capacity for HR repair was associated with increased sensitivity to PARPi and the combination of PARPi and cisplatin caused significantly greater DNA damage compared to cisplatin alone [72].

Studies of patients with other types of cancer suggest value in measuring PARP expression or activity. In a retrospective study of paraffin-embedded tissue samples from patients with resected stage II and III gastric cancer, IHC staining was performed for MLH1, MSH1 ARID1A, PARP-1, BRCA1 and ATM. PARP-1 was found to be the most commonly mutated DNA damage response (DDR) protein. Patients who underwent surgery and whose tumors showed low expression of PARP-1 exhibited a shorter overall survival than that of those with high expression. This retrospective study suggested that PARP-1 expression could be of prognostic value in select solid tumor models [73]. Other studies of PARP expression in locally advanced breast cancer and pancreatic cancer also reported that low expression of PARP-1 was associated with poor prognosis [74,75]. Similarly to other biomarkers, PARP-1 expression was reported with varying scoring systems between studies. Additionally, the studies examining PARP-1 in locally advanced breast cancer and pancreatic cancer were relatively small, bringing into question its generalizability to another cancer population such as UC. A study of patients with high-grade ovarian cancer treated with adjuvant platinum-based chemotherapy, the progression-free and overall survival was significantly better for patients whose tumors showed low PARP expression. This data suggests that PARP expression could be of predictive value for patients treated with cisplatin [76]. Finally, a retrospective study of patients with locally advanced breast cancer found that high levels of PARP-1 expression in pretreatment biopsies was associated with a lower lymph node stage and longer overall survival [74].

A limited number of studies of PARP expression or activity have been conducted using patients with MIBC. Some phase II clinical trials have reported that the presence of genomic alterations in DDR pathways is predictive of a response to neoadjuvant cisplatin chemotherapy. One trial of neoadjuvant MVAC showed that the presence of alterations in *ATM*, *RB1* or *FANCC* was associated with the achievement of a complete pathologic response and predictive of improved PFS and OS [77]. A subsequent phase II trial reported that mutations in *ERCC2* correlated with a pathologic complete response after NAC [33]. However, a more recent study did not find correlation between the presence of ERBB2, ATM, RB1 or FANCC mutations and a pathologic response [34]. There remains an unmet need to discover genomic biomarkers that will be of reliable predictive value for MIBC patients treated with cisplatin, and ongoing investigations into PARP in this disease setting may prove a clinically relevant correlation.

A study of 104 patients with metastatic urothelial cancer treated with first-line cisplatin examined the correlation between ERCC1, RAD51, PARP-1, PAR, BRCA1 and BRCA2 with overall survival. Decreased expression of PAR (but not PARP-1) was associated with better overall survival [78]. A small randomized phase II trial of the PARP inhibitor rucaparib given as amaintenance therapy following cisplatin, was shown to improve progression-free survival for patients with various DDR mutations [79]. A second randomized phase II trial for cisplatin-ineligible urothelial cancer patients showed that the addition of olaparib to durvalumab did not improve the progression-free survival of intent-to-treat population but did prolong the progression-free survival of those patients with HRR mutations [80]. A small single-arm phase II trial of olaparib in UC patients harboring DDR gene alterations showed no objective responses in 19 patients. However, as the authors point out, this apparent negative result may have occurred because of cross-resistance with platinum-resistant disease, as 17 of the 19 patients enrolled previously experienced disease progression after prior platinum therapy. In addition, the limited clinical activity may have resulted because of small sample size, the inclusion of patients with genomic variants of uncertain significance, the measurement of genomic alterations based upon archived primary tumor specimens and brief exposure to Olaparib [81].

Few studies have examined the potential predictive and prognostic value of PARP expression or activity in MIBC patients treated with neoadjuvant cisplatin-based chemotherapy. Few have evaluated the efficacy of PARP inhibitors in patients previously treated with cisplatin. Low PARP-1 expression and/or activity may have a role as a predictive biomarker for a response to neoadjuvant or adjuvant cisplatin. Presently, several active phase 1 and 2 clinical trials are designed to evaluate the potential value of PARP inhibitors in the treatment of urothelial cancer both in metastatic and neoadjuvant settings [82].

## 9. Circulating Tumor DNA (ctDNA)

**Figure 4:**
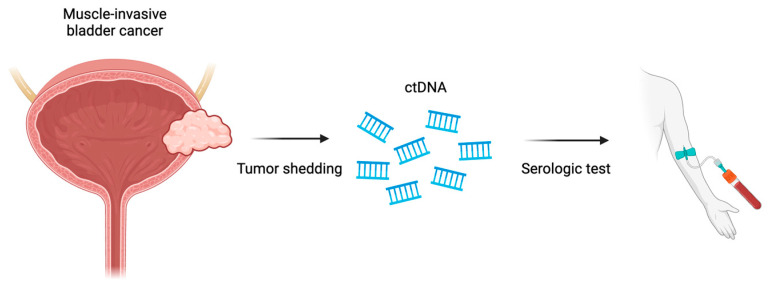
Potential clinical application of ctDNA as a serological test which is a result of tumor shedding.

Circulating tumor DNA (ctDNA) can be assayed as fragments of DNA shed into the bloodstream as a result of cellular apoptosis and the release of cellular products from tumor cells [83,84]. The presence of active malignancy has been associated with elevated ctDNA levels. Because of short half-life, the measurement of ctDNA allows for real-time information regarding the status of tumor burden and the detection of tumor-specific genomic alterations (gAs) [85,86,87]. As a result, ctDNA has been termed the “liquid biopsy” and has proven value in clinical practice, especially in urothelial carcinoma [88]. Invasive procedures, such as cystoscopy, cystectomy and the biopsy of metastatic sites remain the gold standard. However, tissue approaches utilized for diagnosis, surveillance and response assessment require invasive procedures. These procedures may place the patient at risk, may not allow for quantification by serial measurement and have been associated with long turnaround times [88,89]. However, ctDNA analysis allows for noninvasive, rapid acquisition of disease characterization including NGS, and early studies show clinical application in urothelial carcinoma (Figure 4) [85,87,89,90].

The utility of ctDNA as a diagnostic tool in UC has been studied with mixed results. In three studies with a total of 89 patients with metastatic UC (mUC), 73% were noted to have detectable ctDNA levels. A more recent cohort study of 369 patients with metastatic UC detected ctDNA levels in 90% and 95% of patients with lower- and upper-tract UC, respectively [91]. However, the detection of ctDNA levels is significantly lower in patients with localized UC and was detected in only 14% of the patients studied overall [92,93,94]. Current limitations in the detection of ctDNA in patients with localized or non-muscle-invasive bladder cancer limit the application of this tool for surveillance or screening.

However, a potential alternative is the use of urinary assays to detect urine tumor DNA (utDNA), which, like ctDNA, is derived from tumor cell products shed into the urinary tract. An animal model showed the potential utility of this assay [95]. In a clinical study of 118 patients with NMIBC, utDNA was detected in 91% of patients who developed recurrent disease and the detection of utDNA preceded clinical disease recurrence in 92% of patients by a median of 2.7 months. The performance of this utDNA assay exceeded that of commonly used tests including UroVysion, cytology and cystoscopy [96]. It detected 100% of cases found by cytology and detected 82% of cases missed by cytology. This assay appears to be a promising approach for the early detection and surveillance of bladder cancer but requires prospective validation in patients with NMIBC disease.

Despite a limited role in diagnosis and screening, the use of ctDNA has demonstrated greater clinical utility in determining the risks of recurrence for patients undergoing cystectomy and determining the response to therapy for patients with recurrent disease. In the neoadjuvant setting, detectable ctDNA levels in patients with muscle-invasive bladder cancer prior to and after neoadjuvant chemotherapy treatment have been shown to reflect disease persistence and recurrence, while undetectable levels prior to chemotherapy and the clearance of ctDNA following chemotherapy were associated with a lower risk of recurrence [97,98,99]. This suggests that the measurement of ctDNA could serve a prognostic role in complementing the traditional TNM staging system in making treatment decisions [99].

Similarly, the post-surgical detection of ctDNA was associated with disease recurrence in 75% of patients and shown to predate radiographic evidence of disease recurrence by 96 to 243 days. Higher post-operative levels were shown to be associated with metastatic relapse or disease progression compared to undetectable ctDNA levels in those with disease remission [97,98,100]. Elevated levels of ctDNA levels have been associated with worse overall survival [100,101].

Interestingly, quantitative changes in ctDNA levels have demonstrated evidence to support treatment efficacy in UC, namely in adjuvant therapy, as patients noted to have undetectable or rapidly decreasing levels of ctDNA were associated with treatment response [98,102]. In a phase III trial of 581 patients with muscle-invasive bladder cancer, patients treated with adjuvant atezolizumab who demonstrated the clearance of ctDNA levels were noted to have a strong treatment response with disease remission and increased overall survival [103]. Similarly, in a phase I trial of 18 patients treated with pembrolizumab and radiotherapy, a response to treatment was associated with a declining level of ctDNA, while patients with stable or increasing ctDNA levels were shown to be non-responsive to therapy [102].

The role of ctDNA in assessing prognosis and response to treatment shows promising clinical potential and is the subject of phase II/III clinical trials (IMvigor011/NCT04660344, NCT04660344) [103]. ctDNA as a biomarker appears to have a strong potential to impact clinical decision making, but at the present time quantification of the assay has not been standardized and concordance between different platforms has not been evaluated. Further validations are needed before ctDNA can be accepted to prognosticate the risk of recurrence, to select appropriate patients for adjuvant therapy, to monitor response to treatment and to use as a marker for the surveillance of patients following treatment.

## 10. Conclusions

The treatment landscape for urothelial carcinoma is rapidly evolving. Platinum-based chemotherapy remains a backbone of treatment for UC; however, immunotherapy and targeted therapies with antibody–drug conjugates and small-molecule inhibitors now have increasingly important role as standard therapies. The emergence of novel therapies and treatment combinations brings a complexity to the treatment paradigm within UC. Responses to therapy vary tremendously and toxicity is ubiquitous. As such, there is an ever-pressing need for predictive and prognostic biomarkers. We have reviewed many of the biomarkers that may help further delineate our treatment algorithms (Table 1). Identifying those who would benefit from specific treatment strategies would invariably lead to improved outcomes. Additionally, increasing access to reliable testing with blood-based tests would lead to an expedited pathology from the lab to the clinic, with more nuanced clinical decision making. As it stands, many of the biomarkers reviewed were based on samples obtained from tumor tissue, making routine use difficult and limited based on access to technology. The existing biomarkers are fairly limited in their use with only PD-L1 and FGFR having been validated for use with selecting therapies. Many of the markers reviewed are not the standard of care yet and require further study with larger patient cohorts. The markers we have discussed continue to show promise and with more dedicated study will hopefully lead to more standard-of-care testing.

## Figures and Tables

**Table 1:** Summary of prognostic and predictive biomarkers in urothelial carcinoma.

Marker	Marker Value	Comment
PD-L1	Prognostic Predictive	Inconsistent correlation with worse OS Weak predictive value for response to CPI
ERCC1	Prognostic Predictive	Lower levels associated with longer OS Inconsistent correlation with response to cisplatin
ERCC2	Prognostic Predictive	Inconsistent correlation with OS Lower levels associated with response to cisplatin
TMB	Prognostic Predictive	No demonstrated correlation with OS Correlation with response to CPI
DDR genes (ATM, RB1, FANCC, MRE11)	Prognostic Predictive	Trend toward longer OS Correlation with ORR to cisplatin +/− radiotherapy
FGFR3	Predictive	Predictive of response to FGFR inhibitors
HER2/neu	Prognostic Predictive	Limited data—does not appear to have prognostic or predictive value
EGFR (HER1)	Prognostic Predictive	Limited data—does not appear to have prognostic or predictive value
PARP	Prognostic Predictive	Not yet shown to be predictive for OS or response
ctDNA	Prognostic Predictive	Elevated ctDNA levels predict clinical recurrence in patients treated by cystectomy Not shown to be predictive for response

CPI = check-point inhibitor; ctDNA = circulating tumor DNA; OS = overall survival.

## Data Availability

No new data were created or analyzed in this study. Data sharing is not applicable to this article.
